# Novel Live Alkaline Phosphatase Substrate for Identification of Pluripotent Stem Cells

**DOI:** 10.1007/s12015-012-9359-6

**Published:** 2012-03-18

**Authors:** Upinder Singh, Rene H. Quintanilla, Scott Grecian, Kyle R. Gee, Mahendra S. Rao, Uma Lakshmipathy

**Affiliations:** 1 Molecular Probes, Life Technologies, 29851 Willow Creek Road, Eugene, OR 97402 USA; 2 Primary and Stem Cell Systems, Life Technologies, 5791 Van Allen Way, Carlsbad, CA 92008 USA; 3 Present Address: National Institutes of Health-Center for Regenerative Medicine, Bethesda, MD USA; 4 Life Technologies, 5781 Van Allen Way, Carlsbad, CA 92008 USA

## Introduction

Alkaline phosphatases (AP) are a class of enzymes that hydrolyze phosphate containing molecules under alkaline conditions. In humans, there are primarily four different types of this enzyme; intestinal, placental, placental-like and non-tissue specific forms. The non-tissue specific isozyme of AP is expressed in liver, bone and kidney. A similar isozyme was identified in pluripotent stem cells when monoclonal antibodies, TRA-2-49/6E, recognizing determinants of human embryonal carcinoma (EC) cells showed specific reactivity to this isoform.^1^ AP is also known to be expressed at high levels in other pluripotent stem cell types such as embryonic germ cells (EG), embryonic stem cells (ESC) and induced pluripotent stem cells (iPSC).^2^–^5^ Although definitive measures of pluripotency involves *in-vitro* tri-lineage differentiation and *in-vivo* teratoma formation, the most widely tested and validated panel for initial evaluation of ESC and iPSC consists of *S*tage *S*pecific *E*mbryonic *A*ntigen SSEA4; *T*umor *R*ejection *A*ntigens TRA-1-60, TRA-1-81; AP; Oct4 and Nanog.^6^, ^7^

In the case of murine ESC, AP positive colony forming *in-vitro* assay is used as a measure of pluripotency to demonstrate the ability of cells to single cell clone, attach and proliferate.^8^ A similar assay has been adapted for hESC where the sensitivity of the AP positive Colony forming assay to detect loss of pluripotent hESC has been found to be more sensitive than marker expression.^9^ More recently, the onset of AP positive colonies during early stages of reprogramming is used as an initial indicator of successful reprogramming of cells. Furthermore, in some instances the number of AP positive colonies is used as a mark of reprogramming efficiency.^10^ Nevertheless, this marker alone is not a definitive marker for the established iPSC clones. Additional marker evaluation is necessary to identify and qualify bona fide iPSCs.^11^ AP expression levels is a less sensitive measure to differentiate between undifferentiated and early differentiating cells since its expression level is reported to be varied depending on the lineage of differentiation.^12^ AP staining has been used as a fast and easy method that results in a specific chromogenic or fluorescent staining of the pluripotent stem cells. However, the current methods using AP staining require cell fixation and/or result in end products that accumulate within the cells. As a result, these AP stained colonies often lose their morphology and cannot be propagated any further. Inability to further culture selected pluripotent colonies identified using AP staining is a serious disadvantage of this methodology. An ideal solution would be an AP substrate that stains cells without altering the integrity or characteristics of stem cells thereby allowing further expansion of the stained colonies.

Here in, we report the development and application of a novel fluorogenic live cell permeant substrate for AP (Live AP Stain). When incubated with cells for 20–30 min in basal culture media, this stain shows specific and robust staining of pluripotent cells such as human EC, murine and human ESC and iPSC with minimal or no staining of feeder cells and human fibroblasts. Stained colonies retain their morphology and preserve their cell health. The green fluorescence of the stained colonies is eliminated from cells 60–90 min after removal of the stain from the media. We have further utilized this stain in iPSC work flow to identify emerging iPSC clones during reprogramming of BJ human fibroblasts using CytoTune™; a Sendai-virus based non-integrating reprogramming method.^13^ Clones with robust AP staining were manually picked and propagated further. Expanded clones expressed other pluripotent markers, differentiated into cell types representative of the three germ layers and maintained a normal karyotype. These results indicate that AP Live Stain reported in this study does not alter the integrity or characteristics of the stained cells and is therefore an ideal tool to label early intermediates during iPSC generation or clonal populations of ESC for further selection and expansion.

## Materials and Methods

All reagents purchased from Life Technologies, unless otherwise noted.

### Cells

NTERA-2 cl.D1 (ATCC) and BJ human fibroblasts (ATCC) purchased from ATCC, were cultured as per recommendations.

### hESC and hiPSC Culture

Feeder-dependent human H9 ESC (WA09, WiCell Research Institute) and internally generated hiPSC were cultured in hESC media comprising of DMEM/F-12 media containing 20% KnockOutTM SR (KSR), 10 µM MEM Non-Essential Amino Acids solution, 55 µM 2-Mercaptoethanol and 4 ng/ml basic FGF on mitotically inactivated mouse embryonic fibroblast (MEF) feeder layer and maintained in a 5% CO_2_, 37°C, humidified incubator. For feeder-free culture, hESC and hiPSC were cultured in StemPro® hESC SFM, supplemented with 100 µM 2-Mercaptoethanol and 8 ng/ml basic FGF grown on Geltrex®, hESC-qualified reduced growth factor basement membrane matrix coated onto tissue culture treated surfaces. hESC and hiPSC were routinely passaged either using Collagenase IV or the StemPro® EZPassage™ disposable stem cell passaging tool. hiPSC clones were mechanically picked and propagated during the early stages of reprogramming.

### Reprogramming

BJ human neonatal foreskin fibroblast cells (ATCC) were seeded at appropriate plating densities onto TC treated dishes and transduced with the CytoTune™-iPSC reprogramming kit overnight as per the product manual. Seven days post-transduction, cells were harvested in single cell suspension and re-seeded at the desired densities onto MEF feeders in hESC media. Compact colonies with distinct features of pluripotent stem cells started to emerge at around 2 weeks post- transduction. Colonies at 21 days post-transduction were identified based on robust alkaline phosphatase activity using the AP Live Stain. Individual clones were scored using a 27-gauge needle, picked manually and expanded further.

### Preparation of Live Alkaline Phosphatase Stock Reagent

A 10 mM stock solution of the compound was prepared by careful dissolution of the AP Live Stain in tissue-culture grade DMSO. This stock was further diluted 1:100 in DMSO to yield 0.1 mM or 500X stocks (A14353, Life Technologies). These stocks are functionally stable over several months when stored at −20 C without repeated freeze thaw.

### Live Alkaline Phosphatase Staining

ESC and iPSC cultures to be stained for AP activity were washed twice with fresh, sterile, pre-warmed basal DMEM/F-12 media before proceeding. A range of dilutions between 0.25 to 1 X was tested in both negative (BJ human fibroblasts and Murine embryonic fibroblasts) and positive control cells (H9 ESC) and 1X was found to be optimal in providing a robust signal (Supplement Figure 1). For all further experiments 1X working solution was used by diluting the 500X stock solution of the LIVE AP substrate in basal DMEM/F-12 media. Once diluted, the LIVE AP dye was immediately applied on to the adherent cell culture. The cells were incubated with the substrate for 20–30 min and washed twice with the basal DMEM/F-12 media to remove excess substrate. Following the final wash, fresh basal media was added prior to the visualization of fluorescent-labeled colonies under fluorescent microscopy using a standard FIT-C filter. Images were captured within 30–60 min after staining and the most robustly fluorescing colonies were marked for selection and expansion where appropriate. Following visualization, the basal media was replaced by fresh hESC growth media and the selected colonies were either manually picked or returned to the normal culture conditions.

### Antibody Staining

Cells were fixed with 2% paraformaldehyde and blocked with buffer containing 5% Normal goat serum, 1% BSA, 0.1% Triton X100 in dPBS for 30 min. Primary antibodies were added to the cells and incubated at 4°C overnight followed by addition of appropriate secondary antibodies. DAPI was used for counter nuclei staining. Images were captured using a Zeiss Axiovision microscope and images were processed using Adobe Photoshop CS. Antibodies used in this study were: Oct4 (1:500, Abcam), SSEA4 (1:500,), Tra-1-60 (1:100,); Tra-1-81 (1:500,), βIII Tubulin (Tuj1, 1:2000, Sigma), Smooth muscle actin (1:2000, Sigma), α-fetoprotein (1:1000, Sigma), AlexaFluor 488 and AlexaFluor 594 Rabbit anti-mouse IgG (1:500). Alternate AP assays Elf97 and Vector Red (Vector Labs) staining was carried out as per Manufacturer’s protocol.

### Cell Health

H9 ESC was seeded in Geltrex-coated 96 well dishes under feeder-free culture conditions. Cells were treated with experimental conditions in replicates of 6 and were either untreated (Control) or treated with the different lots of AP Live Stain. Conditions known to impact cell integrity such as incubation with 1% paraformaldeylde (PFA) or 50% dimethyl sulfoxide (DMSO) were included as negative controls. Each condition, seeded in 6 replicates was first stained with the AP Live Stain and visually confirmed to be stained positive. Allowing 2 h after stain removal for the stained cells to lose their fluorescence staining, PrestoBlue™ Viability reagent was added to the cells and incubated at 37 C in 5% CO_2_ chamber over night (~20 h). Fluorescence was measured 18–20 h after PrestoBlue™ addition. Average value of the fluorescence reading was measured at 590 nm was plotted with error bars representing standard deviation.

### Karyotype Analysis

Karyotype was carried out by CellLine Genetics. Results were based on cytogenetics analysis performed using GTL-banding technique on twenty G-banded metaphase cells.

### Differentiation

Undifferentiated hESCs and iPSC were harvested using collagenase to generate embryoid bodies (EBs). Cells were cultured in suspension for 4 days in differentiation medium containing DMEM-F12 with Glutamax, 20% KSR, 100 µM MEM-NEAA, and 55 μM 2-mercaptoethanol. On day 5, EBs in suspension were seeded on Geltrex coated plates for an additional 17 days of differentiation in the same medium, then the cells were used for immunocytochemistry.

## Results

Rational design and development was utilized to develop a novel nontoxic AP Live Stain. An ideal nontoxic fluorescent end product was first chosen followed by its phosphorylation to create a phosphatase substrate. Subsequent modifications were carried out to enhance the cell permeability of the phosphatase substrate to create few candidate molecules. The methods, structures and compositions of these molecules are described in a separate study (Manuscript under preparation). A modified phosphorylated green fluorescent dye referred to as “AP Live Stain” was evaluated for pluripotent stem cell detection in this study.

**Fig. 1:**
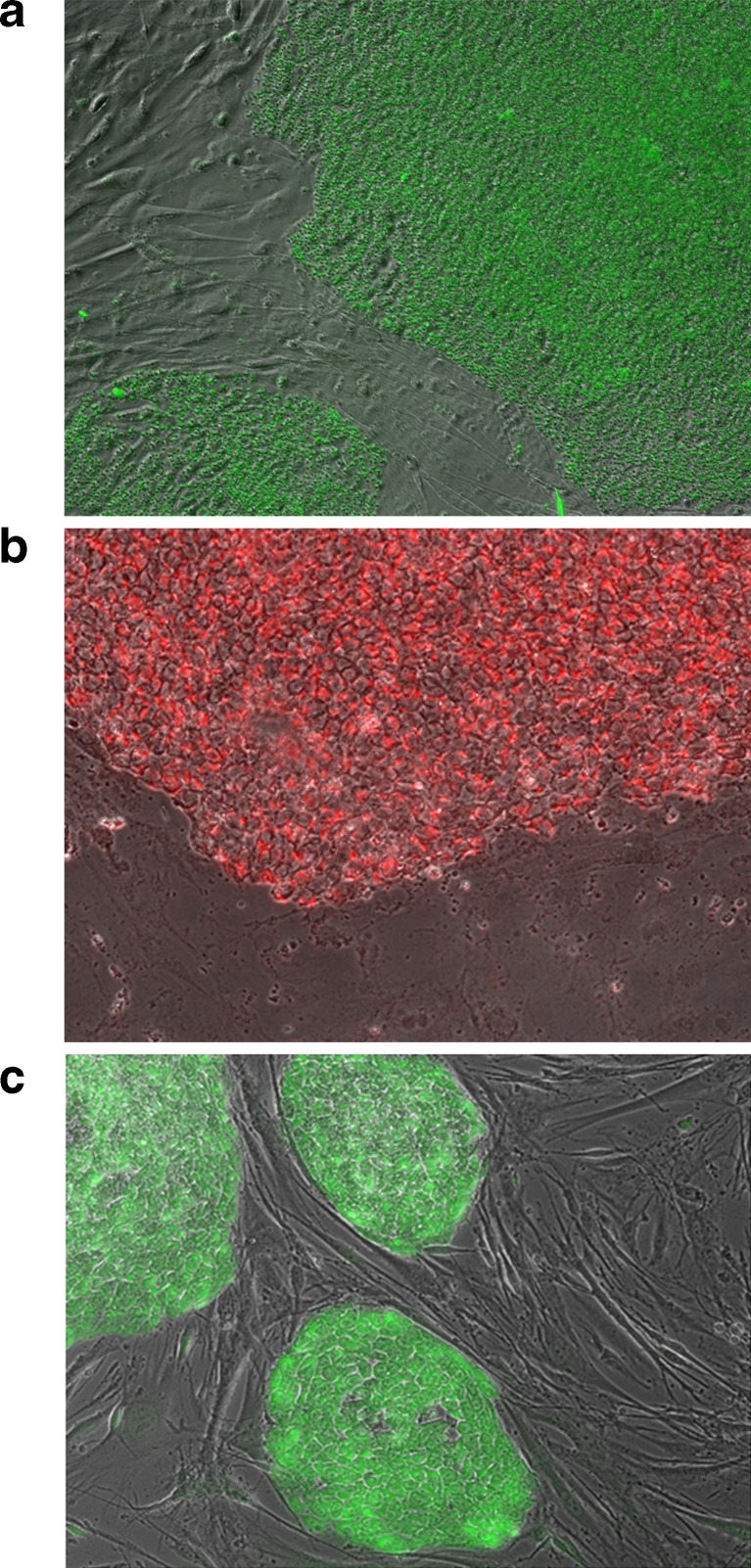
Traditional dyes that are used as substrates to measure elevated alkaline phosphatase activity in pluripotent stem cells, such as ELF97 (**a**) and Vector Red (**b**) result in stained cells that cannot be propagated further. Rational design and synthesis was carried out to develop a AP Live Stain and shown to specifically stain pluripotent stem cells (**c**). The morphology of the fluorescent labeled cells appeared intact in comparison to cells stained with traditional dyes

H9 ESC on feeders was stained with two available commercial products along with the AP Live Stain. Enzyme-labeled Fluorescence 97 (ELF-97) is a soluble phosphorylated molecule that yields green fluorescent precipitate when cleaved by AP. These resulting fluorescent precipitates can be visualized under FITC excitation/emission filter systems using standard fluorescence microscope. The staining is specific to the ESC colony with little or no signal in the surrounding feeder cells (Fig. 1a). Vector® Red yields a highly fluorescent bright red precipitate when cleaved by AP and can be viewed visually or with Texas Red® or Rhodamine excitation/emission filter systems. The staining is bright red, specific to the ESC colony with minimal background (Fig. 1b). Both these reagents are fast and simple to use but require permeabilization and fixation of the cells. The resulting stained colonies lose their integrity and peel off the dish with time, a sign of cellular mortality. Thus, AP assay using these reagents is an end-point assay where cells once stained cannot be expanded further for downstream applications. Live AP Stain on the other hand generates a soluble phosphorylated molecule inside the cell that in presence of AP yields a non-toxic green fluorescent product. This product can be visualized using FITC excitation and emission filter systems. ESC colonies show specific and robust staining while the surrounding MEF feeder cells remain unstained (Fig. 1c). This differs from the ELF-97 and Vector® Red AP stains since the fluorescent product from AP Live Stain does not accumulate in the cells and the stained cells loose the fluorescence as the fluorescent product permeates out of the cells with time.

**Fig. 2:**
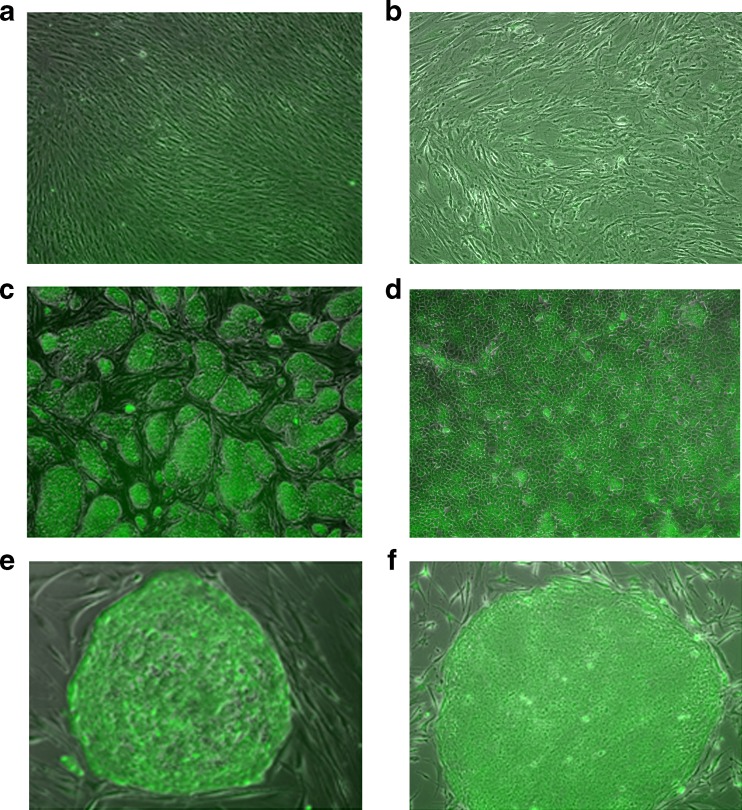
The AP Live stain was incubated with different cell types under identical conditions. Phase contrast and fluorescence images captured using FITC filter were captured using an Axiovert fluorescence microscope. Adobe Photoshop was used to generate overlap images of phase contrast and fluorescence images. **a** BJ human Fibroblast, the most commonly used parental cell line for somatic reprogramming **b** Murine Embryonic Fibroblasts, mitotically inactivated, and commonly used as feeder cells for ESC and iPSC culture **c** 129/SvEv murine ESC cultured on inactivated MEF feeder cells **d** NTERA2 c.D1 human embryonal carcinoma cells **e** Human H9 (WA09) ESC cells cultured on inactivated MEF feeder cells **f** iPSC derived from BJ human fibroblast using CytoTune™ Reprogramming and cultured on inactivated MEF feeder cells

Different types of cells were stained with the AP Live Stain to further demonstrate the specificity of the AP Live Stain. BJ human fibroblast is widely used as the parental cell type for somatic reprogramming using various methods. These cells demonstrated insignificant staining that required long exposure times (Fig. 2, panel A). MEF cells that are commonly used as feeders for pluripotent stem cell in a feeder-dependent culture also exhibited weak staining similar to human fibroblasts (Fig. 2, panel B). On the other hand, the human embryonal carcinoma cell line NTERA2 that is known to have gene expression profiles similar to embryonic stem cells^1^ stained positively with the AP Live Stain (Fig. 2, panel D)

Several different types of stem cells demonstrated robust and specific staining with AP Live Stain under identical incubation conditions. Incubating cultured colonies of 129/SvEv mESC (Fig. 2c) or H9 hESC (Fig. 2e) on a layer of MEF feeder cells displayed robust and specific staining of mESC or H9 hESC over MEF feeder cells. Furthermore, incubating established iPSC lines cultures on feeders that are derived from BJ fibroblasts using CytoTune™ iPS-Sendai Reprogramming method show equally strong AP staining (Fig. 2f). The minor differences noted in intensities between different cells types is largely due to size of the colony with the signal more robust in the larger human ESC and iPSC relative to the smaller mouse ESC or monolayer embryonal carcinoma cells. Additionally, in all cases, the typical morphology of the ESC colonies with high nucleus to cytoplasmic ratio with distinct refractive colony edges were retained after staining with AP Live Stain and was similar to the unstained control.

**Fig. 3:**
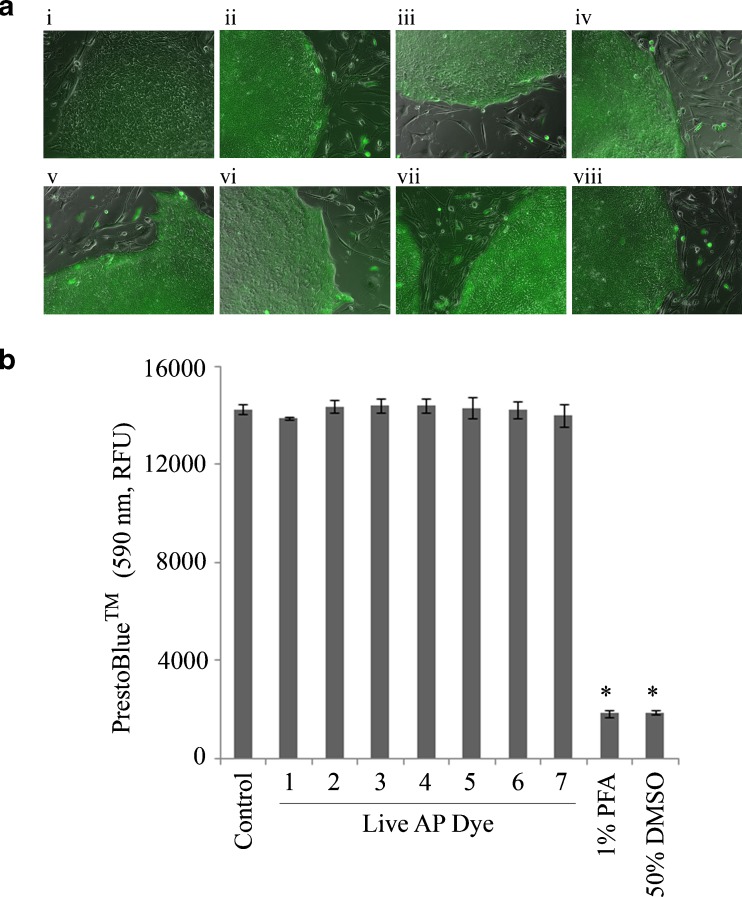
**a** Feeder-dependent H9 ESC cultures (i) were stained with 7 independent preparations of AP Live Stain (ii–viii). Images were collected at 10X magnification following 30 min incubation with AP Live Stain followed by washes. Specific staining of the ESC colonies was observed with all the samples. **b** Cell health of AP Live stained cells was assessed using PrestoBlue™ cell vitality dye. H9 ESC was seeded in 96 wells with 6 replicates for each test conditions. Wells were stained with 7 independent preparations of AP Live Stain. Wells not treated with AP Live Stain served as positive controls while wells treated with known cell disrupting agents such as Paraformaldeyhe (PFA) and Dimethyl sulfoxide (DMSO) served as negative controls. Two hour after staining, PrestoBlue™ was added to the wells and incubated for 20 h. At the end of the incubation period, fluorescence at 590 nm was measured and the average values obtained for each sample plotted as a bar graph with error bars representing standard deviation. * indicates conditions where the values obtained was statistically significant with P values >0.05, relative to Control

The ability of AP Live stained cells to preserve their cellular integrity was further confirmed using PrestoBlue™ Cell Viability Reagent. Metabolically active cells continuously convert the PrestoBlue™ Reagent, from a blue compound with no intrinsic fluorescent value to a red product that is highly fluorescent, thereby increasing the overall fluorescence intensity (at 590 nm) of the media surrounding the cells. Non-viable cells, on the other hand, cannot metabolize the indicator stain resulting in no change in fluorescent intensity at 590 nm. The health of the cell under investigation can therefore be monitored by the change in the fluorescence intensity at 590 nm of the media. H9 ESC on feeders (Fig. 3a, panel i) were stained with independent preparations of AP Live Stain to confirm pluripotent specific staining (Fig. 3a, panels ii–viii). Wells with feeder-free H9 ESC samples were used to assess cell health using PrestoBlue™ cell vitality assay and the results represented as bar graphs (Fig. 3b). Control wells with cells that were not treated with AP Live Stain showed high levels of fluorescence indicating a large number of the cells to be metabolically active. Comparable values were obtained with all samples treated with the AP Live Stain. Statistical comparison between the AP Live stained cells to the positive control showed no significant difference. In contrast, the negative control groups (cells treated with known cell disruptive agents such as PFA and DMSO) showed significantly lower levels of fluorescence indicating significantly lower number of metabolically active cells. In comparison to the control, the difference was statistically significant with p values >0.005, as measured by ANOVA (represented as stars over the bars). These results collectively indicate that AP Live Stain does not alter integrity of stained cells.

**Fig. 4:**
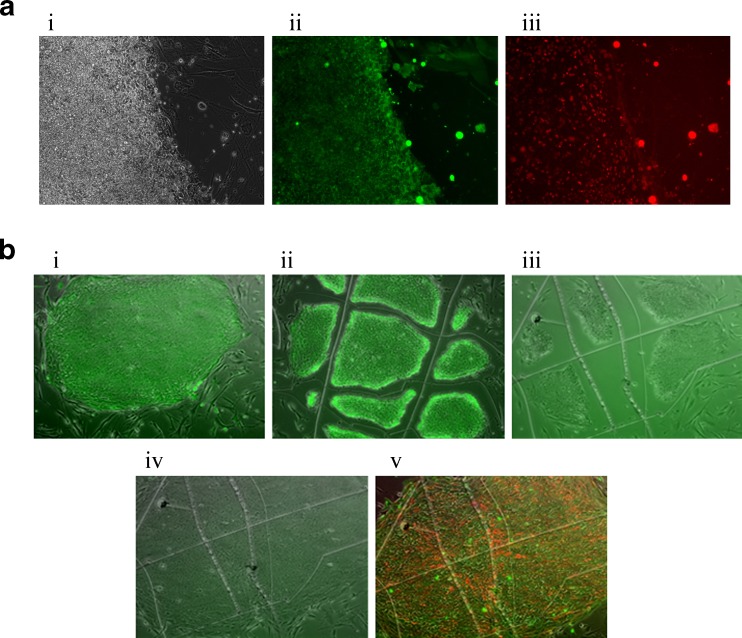
**a** BJ fibroblasts transduced with CytoTune™ reprogramming particles show emergence of colonies that stain positive for AP Live Stain. These colonies also express the pluripotent marker, TRA-1-60. **b** 3 weeks after transduction, the emerging colonies were stained with AP Live Stain and colonies that showed robust fluorescent staining were manually passaged onto feeders for further expansion and characterization. At passage 3, the expanding clones showed distinct ESC-like morphology and stained positive for AP Live Stain (i). The stained colony was scored with a 27 gauge needle and parts of the colony removed. At this stage the scored colonies fluoresce green (ii). 2 h after staining with AP Live Stain and removal of the stain from the growth media, the scored cells do not show any fluorescence (iii). Following recovery of the AP Live stained and scored iPSC colonies for 48 h at 37 C, the scored areas are covered with expanding cells (iv) and the restored areas stained positive for pluripotent surface markers SSEA4 (green) and TRA-1-60 (red) (v)

The utility of AP Live Stain was further evaluated in reprogramming work flow. BJ human fibroblasts transduced with the CytoTune™ iPSC reprogramming particles resulted in formation of colonies 2 to 3 weeks post transduction. Emerging colonies (Fig. 4a, panel i) stained positive with Live AP Stain (Fig. 4a, panel ii), and were also positive for another pluripotent marker Tra-1-60 (Fig. 4a, panel iii). 12 clones that stained positive with AP Live Stain were manually picked and seeded on to fresh dishes with MEF feeders. All of these clones were further expanded to passage 3 at which time six clones were further treated with AP Live Stain.

Figure 4b (panel i) shows a representative image of one of these clones stained with the AP Live Stain. This positively stained clone was scored using a 27 gauge needle during which process parts of the cells around the scoring region was removed and images immediately captured to demonstrate that the iPSC clones labeled with AP Live stain colony had distinct scores with bare regions along the scored edges (Fig. 4b, panel ii). Once the AP Live Stain was removed from the media and the cells allowed to recover for 2 h in normal ESC growth media, the scored colony was again imaged to show loss of the green fluorescence (Fig. 4b, panel iii). These cells, when allowed to recover over 48 h, were able to propagate and phase contrast image of the scored colony shows that the gaps along the scored edges of the colony are now filled with proliferating cells (Fig. 4b, panel iv). As a further confirmation of maintenance of pluripotency in the proliferating areas of the scored clone, all cells within the colony continued to stain positive for the pluripotence surface markers SSEA4 (labeled Green, Fig. 4b, panel v) and TRA-1-60 (labeled Red, Fig. 4b, panel v).

**Fig. 5:**
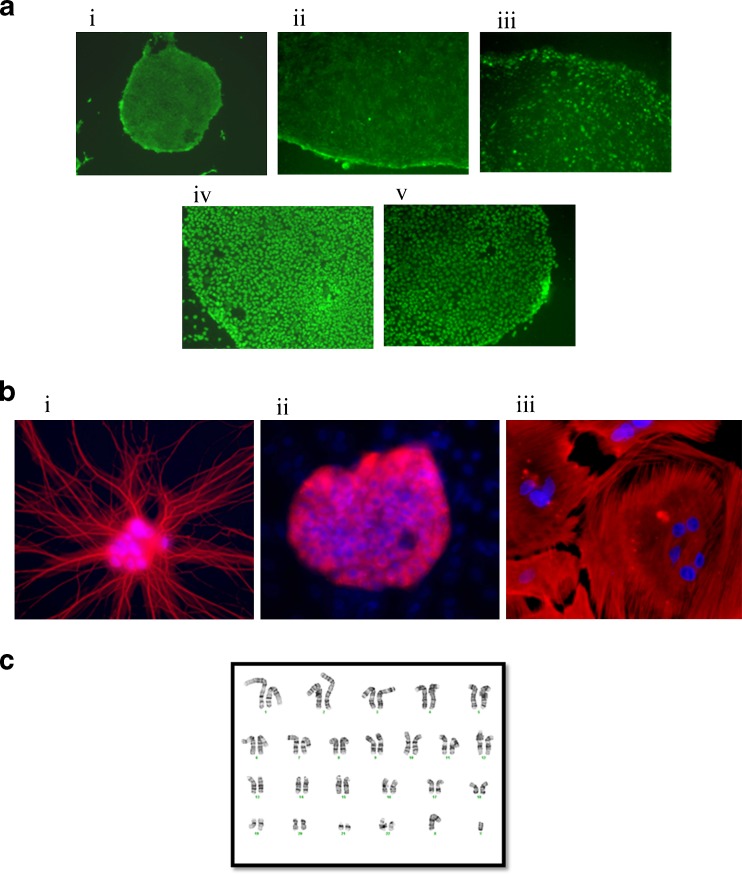
Of the 12 clones initially picked, 3 clones were further expanded and characterized. All clones expressed pluripotent markers, had tri-lineage differentiation potential and maintained a normal karyotype **a** A representative iPSC clone that was initially picked based on robust AP Live staining was expanded to passage 10 under feeder-dependent conditions express pluripotent markers AP (i), SSEA4 (ii), TRA-1-60 (iii), Oct4 (iv) and Nanog (v). **b** Representative iPSC clone was differentiated via random embryoid body formation and at the end of 21 days of differentiation, resulting cells stained positive for lineage specific markers representative of the three germ layers, beta III Tubulin—ectoderm (i), Alpha feto protein—endoderm (ii) and Smooth muscle actin—mesoderm (iii). **c** Cytogenetic analysis performed on twenty G-banded metaphase cells from a representative iPSC clone demonstrated an apparently normal male karyotype (46, XY) and no abnormal cells were detected

Three clones from the above experiment were further propagated to over passage 10 and periodically checked for expression of characteristic pluripotent markers (Fig. 5a): AP Live Stain (panel i), SSEA4 (panel ii), TRA-1-60 (panel iii), Oct 4 (panel iv) and Nanog (panel v). These clones were randomly induced to differentiate via embryoid body formation resulting in various cell types (Fig. 5b). The differentiated cell types stained positively for beta III tubulin an ectoderm neuronal marker (i), alpha feto protein, an endodermal liver marker (ii) and smooth muscle actin, a mesoderm marker. The clone at passage 12 continued to maintain a normal karyotype as determined by G-banding (Fig. 5c). Parallel H9 ESC cultures and iPSC clones that were not treated with AP Live Stain and maintained under similar culture conditions behaved identically to the above clones suggesting no long term impact of AP Live Stain on the cell characteristics.

These results collectively demonstrate that the AP Live Stain is an inexpensive, straightforward and undemanding alternative to existing AP dyes with the added advantage of preserving cell integrity that allows for cell manipulation and proliferation post staining.

## Discussions

Surface antibodies are the most widely used method to specifically identify stem cells in heterogeneous cultures. In particular, SSEA4 and TRA antibodies have emerged as a benchmark for identifying pluripotent stem cells via immunostaining methods. Additional applications of these antibodies include enrichment of the stem cell population via FACS sorting and magnetic bead based separation methods.^14^ Small molecule dyes offer an attractive alternative to antibodies that involve expensive, tedious and time consuming workflows. Aside from workflow issues, potential microbial contamination of antibodies may render cells less useful for some downstream applications. In addition to AP, human embryonic stem cell lines also express drug transporter ABCG2 at high levels.^15^ Rapid efflux of ABCG2 substrates in emerging iPSC cell colonies result in Hoechst^dim^ phenotype that, in combination with expression of other markers, is reported to be an accurate predictor of reprogramming success (11). A recent study screened a large chemical library and identified CDy1, a rosamine analog that specifically stains pluripotent stem cells.^16^ The mode of staining by this dye remains largely unknown, nevertheless, it stains both live human ESC and iPSC and can be used in fluorescence-activated cell sorting.^17^

Although, several of the methods described above offer characterization of ESC and iPSC, a need remains for reagents and methods that could simplify the workflow without any detrimental effects on the cells. An ideal scenario would be to accomplish the characterization of stem cells without leaving any morphological, physiological or chemical ‘footprint’ on them. We envisioned a nontoxic, cell permeant, fluorogenic stain that selectively reports on the activity of AP may meet the desired requirements. In order to meet the no ‘footprint’ requirement, this stain should permeate out of the cells after reporting AP activity. The newly designed AP Live Stain reported in this study meets all of these requirements to characterize iPSC and ESC. Since the resulting reporter fluorescent compound is a small molecule that can permeate out of the cells after a short period of time, the AP Live Stain extends the widely used AP assay from terminal staining assay to an assay for real time monitoring of pluripotent stem cell cultures without compromising cell integrity or requiring duplicate cultures. Besides its use in monitoring reprogramming process to screen and choose emerging colonies, it is also applicable to other work flow such as clonal expansion of cells for creation of genetically manipulated pluripotent stem cells where gross morphological changes are less apparent than marker expression.

Although, the focus of this report has been limited to applications of AP Live Stain for pluripotent stem cell work flow, it has potential use in several different applications. Since AP is an established marker for bone precursor cells, preliminary experiments indicate that the AP Live Stain also stains differentiating osteoblasts derived from mesenchymal stem cells (unpublished results). Thus, AP Live Stain could be utilized in HTS format for screening and indentifying inducers of bone formation. This stain could also serve as a research reagent for identification and detection of differentiating bone and utilized for enrichment of osteoblasts for downstream applications, i.e. 3D bone reconstruction and cell therapy. AP is also a general stem cell marker and has been used to identify in vivo niche of stem cells such as trabecular meshwork in limbal and corneal endothelium.^18^ Besides applications specific to stem cell, it could be used for tracking transgenic cells in transplantation experiments as AP has been used as a marker to indentify these cells.^19^

In conclusion, we have rationally designed and build a nontoxic cell permeant fluorogenic AP Stain with desirable features that enables characterization of ESC and iPSC without leaving a footprint on the cells. Its application could be expanded to several other clinical or diagnostic areas where AP is used as marker.

## Electronic supplementary material

Below is the link to the electronic supplementary material.

**Supplement Figure 1: Dilution range of AP Live Stain.** AP Live Stain was diluted to 0.25 to 1X in DMEM/F12 and directly applied on either BJ fibroblasts, MEFs (murine embryonic fibroblasts) or H9 ESC cultured on MEFs. All cells were handled under identical conditions as described under Materials and Methods. Following the staining protocol, images were captured at 10X objective/10X eye piece under auto exposure settings and images compiled using Photoshop. (TIFF 10859 kb)
