# Supplementary figures and images for: Novel Live Alkaline Phosphatase Substrate for Identification of Pluripotent Stem Cells
Source: Stem Cell Rev. 2012 Mar 18;8(3):1021–9. doi: 10.1007/s12015-012-9359-6 (PMC3412082; doi:10.1007/s12015-012-9359-6)

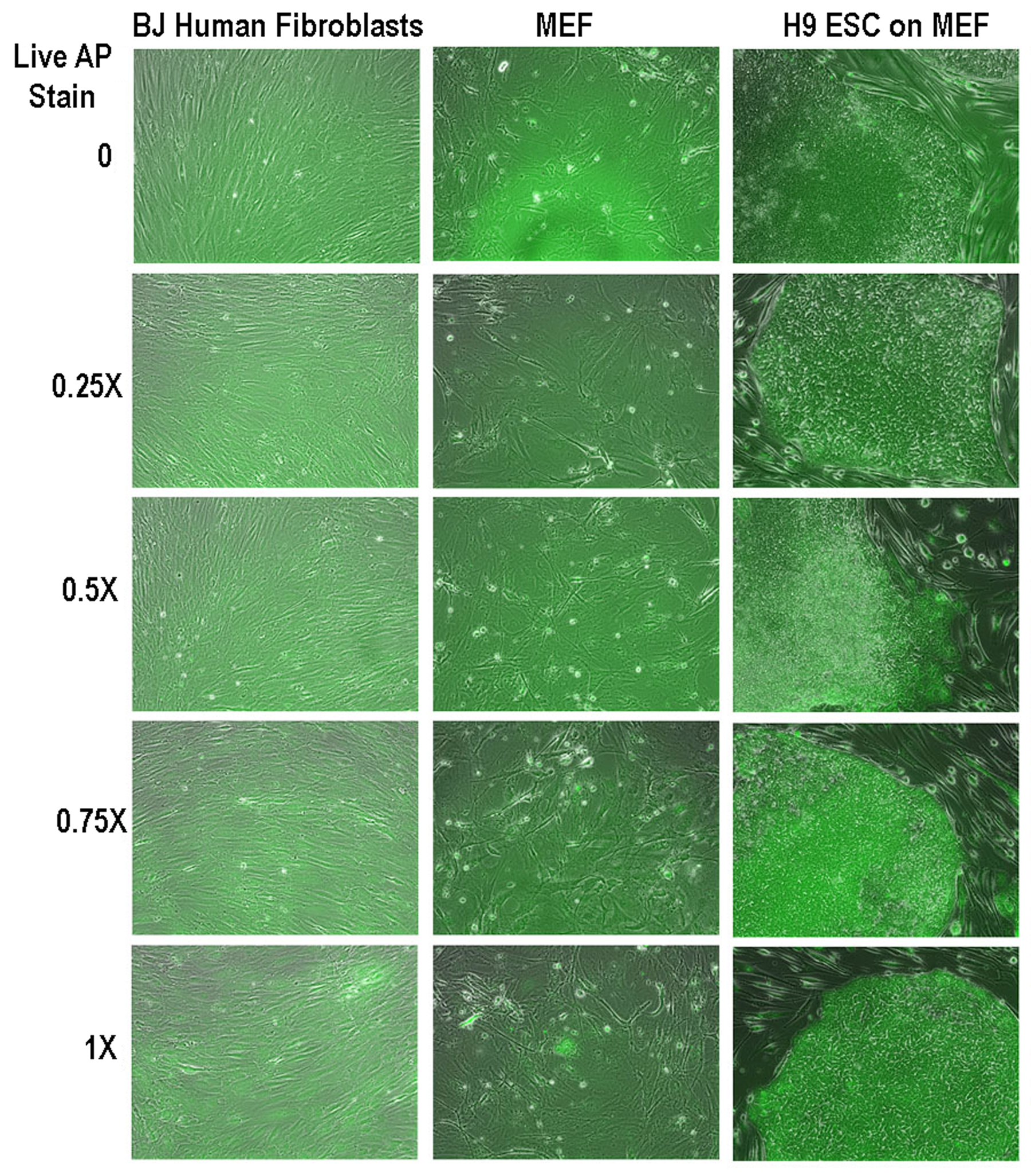

Supplement: Supplementary file 1 — Supplement Figure 1: Dilution range of AP Live Stain. AP Live Stain was diluted to 0.25 to 1X in DMEM/F12 and directly applied on either BJ fibroblasts, MEFs (murine embryonic fibroblasts) or H9 ESC cultured on MEFs. All cells were handled under identical conditions as described under Materials and Methods. Following the staining protocol, images were captured at 10X objective/10X eye piece under auto exposure settings and images compiled using Photoshop. (TIFF 10859 kb) [file 12015_2012_9359_MOESM1_ESM.tif]
